# Enhancer of trithorax/polycomb, Corto, regulates timing of *hunchback* gene relocation and competence in *Drosophila* neuroblasts

**DOI:** 10.1186/s13064-022-00159-3

**Published:** 2022-02-17

**Authors:** Terry L. Hafer, Sofiya Patra, Daiki Tagami, Minoree Kohwi

**Affiliations:** 1 Department of Neuroscience, Mortimer B. Zuckerman Institute Mind Brain Behavior, Columbia University, New York, NY 10027 USA; 2 Present Address: Molecular and Cellular Biology Program, University of Washington, Seattle, WA 98195 USA; 3 Kavli Institute for Brain Science, Columbia University, New York, NY 10027 USA

**Keywords:** Neuroblast competence, Temporal identity, Hunchback, Corto, Polycomb, Nuclear lamina, Nuclear architecture

## Abstract

**Background:**

Neural progenitors produce diverse cells in a stereotyped birth order, but can specify each cell type for only a limited duration. In the *Drosophila* embryo, neuroblasts (neural progenitors) specify multiple, distinct neurons by sequentially expressing a series of temporal identity transcription factors with each division. Hunchback (Hb), the first of the series, specifies early-born neuronal identity. Neuroblast competence to generate early-born neurons is terminated when the *hb* gene relocates to the neuroblast nuclear lamina, rendering it refractory to activation in descendent neurons. Mechanisms and trans-acting factors underlying this process are poorly understood. Here we identify Corto, an enhancer of Trithorax/Polycomb (ETP) protein, as a new regulator of neuroblast competence.

**Methods:**

We used the GAL4/UAS system to drive persistent misexpression of Hb in neuroblast 7–1 (NB7-1), a model lineage for which the early competence window has been well characterized, to examine the role of Corto in neuroblast competence. We used immuno-DNA Fluorescence *in situ* hybridization (DNA FISH) in whole embryos to track the position of the *hb* gene locus specifically in neuroblasts across developmental time, comparing *corto* mutants to control embryos. Finally, we used immunostaining in whole embryos to examine Corto’s role in repression of Hb and a known target gene, Abdominal B (Abd-B).

**Results:**

We found that in *corto* mutants, the *hb* gene relocation to the neuroblast nuclear lamina is delayed and the early competence window is extended. The delay in gene relocation occurs after *hb* transcription is already terminated in the neuroblast and is not due to prolonged transcriptional activity. Further, we find that Corto genetically interacts with Posterior Sex Combs (Psc), a core subunit of polycomb group complex 1 (PRC1), to terminate early competence. Loss of Corto does not result in derepression of Hb or its Hox target, Abd-B, specifically in neuroblasts.

**Conclusions:**

These results show that in neuroblasts, Corto genetically interacts with PRC1 to regulate timing of nuclear architecture reorganization and support the model that distinct mechanisms of silencing are implemented in a step-wise fashion during development to regulate cell fate gene expression in neuronal progeny.

**Supplementary Information:**

The online version contains supplementary material available at 10.1186/s13064-022-00159-3.

## Background

In both insects and mammals, neural progenitor cells generate diverse cell types in a stereotyped birth order [1–7]. Throughout development, progenitors progressively lose competence to generate early-born neural cell types as they gain competence to generate the late-born cell types, thereby establishing organized tissue growth. Thus, progenitors have a limited window of time during which they are able to specify each neural cell type [8–12]. How competence is regulated is critical to understanding both normal development and for effectively harnessing stem cells therapeutically, but mechanisms underlying competence regulation are not well understood.

*Drosophila* neural progenitors, called neuroblasts, are a tractable model system to investigate mechanisms of competence regulation. In the embryonic ventral nerve cord (VNC), 30 bilaterally symmetric neuroblasts undergo repeated rounds of asymmetric divisions, producing a smaller ganglion mother cell that divides once again to generate postmitotic neural progeny. With each division, neuroblasts sequentially express a series of transcription factors, called temporal identity factors, Hunchback (Hb) ➔ Kruppel (Kr) ➔ Pou domain transcription factors 1 and 2 (collectively called Pdm) ➔ Castor (Cas), which specify the neural identity of the descendent neurons [2, 13–18]. While neuroblasts express each temporal identity factor only transiently, the neurons descended from each neuroblast division maintain active expression of the temporal identity factor, thus becoming molecularly marked by the timing of their birth.

**Fig. 1:**
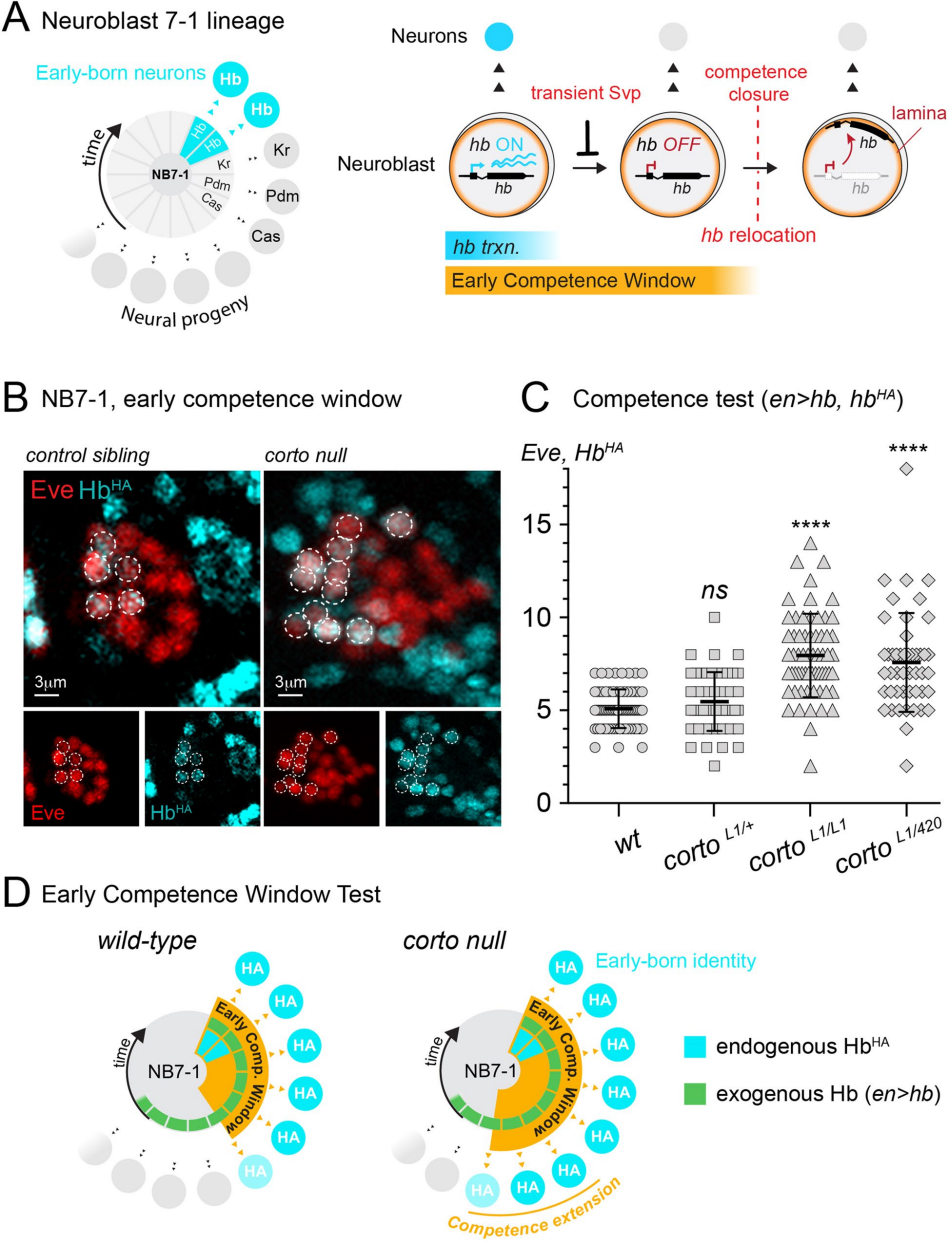
Early competence window is prolonged in *corto* mutants. **A** *Left*, NB7-1 lineage with neural progeny displayed by birth order, clockwise. The neuroblast is at the center and divided into time segments during which each temporal identity factor is expressed. *Right*, *hunchback* (*hb*) is transcriptionally repressed by transient pulse of Svp. Several divisions later, the *hb* gene relocates to the nuclear lamina, rendering the gene refractory to activation in the neural progeny, closing the early competence window. **B** NB7-1 lineage early competence assay in representative *corto* mutant (*corto*^*L1*^) compared to sibling (heterozygous) control embryo. **C** Quantification of early competence window (Eve^+^HA^+^). Genotypes shown on x-axis. Each datapoint represents the average number of early-born (Eve^+^HA^+^) neurons from a single NB7-1 lineage. A minimum of three embryos were quantified per genotype. Data are represented as mean ± SD. P values represent comparisons to wild type. **D** Schematic summary of *corto* mutant competence phenotype

Hb, the first of the temporal identity factor series, is a zinc-finger transcription factor that specifies early-born neural fate in multiple neuroblast lineages, similar to its mammalian homolog Ikaros, which promotes early-born identity in both the retina and the cortex [19, 20]. In neuroblasts, Hb is expressed for only the first one or two divisions and is rapidly repressed by a transient expression of its repressor, Seven-up (Svp) [21, 22]. Though neuroblasts express Hb only briefly, they remain competent to specify *hb*-transcribing early-born neurons for several additional divisions after *hb* is repressed in the neuroblast [9, 11, 12, 23]. Thus, the “early competence window” is a representation of neuroblast potential to generate early-born neurons that are marked by endogenous *hb* transcription, and this potential can be experimentally measured by identifying the neural progeny generated upon misexpressing Hb in the neuroblast progenitor. Previously, we showed that the early competence window is terminated when the *hb* gene physically relocates within the neuroblast to the nuclear lamina [11], a gene silencing hub [24, 25]. This movement to the nuclear lamina occurs much after *hb* is already transcriptionally silent, and renders the *hb* gene refractory to activation in the descendent neurons. Thus, while transcriptional repression by Svp terminates *hb* expression within the neuroblast, physical relocation of the *hb* gene to the nuclear lamina establishes a heritably silent gene state that terminates early competence (Fig. 1A). Little is known regarding the mechanisms underlying this second level of *hb* silencing.

Here we identify an ETP protein Corto as a novel competence regulator. Loss of Corto results in a delay in *hb* gene relocation to the neuroblast nuclear lamina and prolongs competence to specify early-born neural identity. Furthermore, consistent with function as an ETP protein, we found that Corto genetically interacts with Psc, a core subunit of PRC1, to terminate early competence. While polycomb (PcG) chromatin factors are well known for their role in the maintenance of gene repression of its target genes during development [26–28], loss of Corto does not result in derepression of *hb* in late-stage neuroblasts. Together, our results provide insight into new regulators in nuclear architecture and the timing of neural progenitor competence transitions during development.

## Materials and methods

### Fly lines

Wild-type *(w1118)*, *corto*^*L1*^, *corto*^*420*^ (kind gift from Dr. Frédérique Peronnet, [29]), *Psc*^*h27*^ (Bloomington, BL#5547), *en-GAL4* (chrom II, [16, 30], *UAS-hb* [31], *hb*^*HA*^ [11], UAS-corto [kind gift from Dr. Frédérique Peronnet, [29]]. Flies were raised on a standard cornmeal and molasses medium at 25C.

### Immunostaining and antibodies

We immunostained embryos following standard protocols [32]. Briefly, we fixed embryos in a one-to-one mixture N-heptane and 4% formaldehyde diluted in PEM buffer (0.1 M Pipes, 1 mM MgS0_4_, 2 mM EGTA) and rocked them in at room temperature for 22 min. The fixative solution was subsequently removed, and embryos were devitellinized by vigorous shaking in a 1:1 mixture of methanol:heptane. Devitellinized embryos were washed with PBS and incubated overnight at 4 °C in primary antibodies diluted in PBS-0.1% Tween 20 (PBT). After washing, embryos were incubated at room temperature in secondary antibodies for 1.5 h, and streptavidin for 20 min. The following primary antibodies were used: Mouse anti-Eve (clone 3C10, 1:50, Developmental Studies Hybridoma Bank (DSHB)), rat anti-HA (#11,867,423,001, clone 3F10, 1:500, Sigma), rabbit anti-lamin (1:2000, kind gift from Dr. Paul Fischer); rat anti-worniu (#196,362,clone 5A3AD2, 1:200, Abcam), rat anti-dpn (#195,172, clone 11D1CH11, 1:200, Abcam), mouse anti-PH3 (#14,955, 1:1000, Abcam), rabbit anti-Hb (1:200, kind gift from Dr. Chris Doe), rat anti-Zfh2 (1:200, kind gift from Dr. Chris Doe), mouse anti-AbdB (clone 1A2E9, 1:30, DSHB). Secondary antibodies against mouse, rabbit, and rat were conjugated to Alexa 488, 555, and 647 (Thermo Fisher Scientific).

### Immuno-DNA FISH

DNA *in situ* hybridization (DNA-FISH) was performed as previously described [11, 23, 33]. Briefly, embryos were fixed in freshly-prepared 4% formaldehyde diluted in PIPES buffer (60 mM KCl, 15 mM NaCl, 0.5 mM spermidine, 0.15 mM spermine, 2 mM EDTA, 0.5 mM EGTA, 15 mM PIPES pH 7.4) and rocked for 25 min at room temperature with an equal volume of heptane. Embryos were devitellinized by vigorous shaking in a one-to-one solution of methanol:heptane. We generated fluorescent probes by PCR-amplifying approximately 10 kb of the *hb* genomic locus [11] and using the DNA FISH Tag kit (Thermo Fisher Scientific). After rehydration, embryos were treated with RNaseA (150 mg/ml) for 2 h at room temperature and gradually stepped into 100% pre-hybridization solution (pHM: 50% formamide; 4XSSC; 100 mM NaH2PO4, pH 7.0; 0.1% Tween 20). After one hour of pre-hybridization at 37 °C, embryos were denatured 15 min at 80 °C and incubated with denatured FISH probe diluted in pre-hybridization solution overnight at 37 °C in hybridization buffer (10% Dextran sulfate, 50% deionized formamide, 2XSSC, 0.5 mg/ml Salmon Sperm DNA). Embryos were washed at 37 °C in a series of formamide/0.3% CHAPS solutions with decreasing formamide at each wash. After the *in situ* hybridization protocol was completed, embryos were subsequently immunostained according to standard immunochemistry protocol (see above), mounted in Vectashield (Vectorlabs) and imaged on a confocal microscope, Zeiss LSM 700. Additional protocol details are available upon request.

### Confocal imaging

We used a Zeiss 700 Axio Imager 2 laser scanning confocal for all images. DNA-FISH images were taken at a 0.4 µm step size, with pinholes that were adjusted to have equal optical section thickness in all channels.

### Quantification and statistical analyses

We used standard t-tests using Prism v8. Statistical significance was classified as: ** < 0.01, *** < 0.001, **** < 0.0001. Embryos stage 14–16 were used in competence window tests. For graphs in which n’s represent individual neuroblasts, a minimum of three embryos were quantified per genotype, and for graphs in which n’s represent average of single embryos, at least twelve lineages were averaged per embryo. For DNA FISH, n’s represent each embryo, and number of FISH signals quantified are shown on the graph. Graphs show each data point used for statistical analysis, and bars indicate mean ± standard deviation.

## Results

### Loss of Corto extends the early competence window

Neuroblasts undergo two temporally-separated, distinct types of *hb* gene repression. In the first, *hb* is transcriptionally repressed by Svp after one-to-two divisions. In the second, several hours and divisions later, the already transcriptionally repressed *hb* gene physically relocates to the neuroblast nuclear lamina. This relocation causes heritable gene silencing and renders the *hb* gene refractory to activation in the descendent neurons, thereby closing the early competence window (Fig. 1A) [11, 23]. Thus, while neuroblasts typically generate one to two early-born neurons, they are capable of producing more until nuclear architecture reorganization terminates competence. We recently identified the PcG factors as critical players in the second step, in terminating neuroblast early competence. PcG proteins are chromatin factors best known for their function to regulate gene repression through the cooperative activity of two multimeric complexes, PRC1 and PRC2 [26, 28, 34–39]. We found that Psc is required for timely *hb* gene relocation to the neuroblast nuclear lamina and closing the early competence window [23].

Another class of proteins, the ETP proteins, has been shown to interact with the Trithorax and PcG complexes and regulate target genes. Corto, one such ETP protein, has been shown to genetically interact with PcG factors in Hox gene repression [29, 40, 41] and colocalizes with the majority of Psc-bound polytene chromosome sites [42]. Thus, we hypothesized that Corto may also function in regulating neuroblast competence. To test this, we focused on the NB7-1, one of the thirty neuroblast lineages of the embryonic VNC, for which we have markers to identify the progeny as well as have detailed knowledge of the temporal progression of its lineage and competence [9, 11, 12, 16, 23]. Briefly, we took advantage of the GAL4/UAS system [43] and used the *engrailed* GAL4 (*en-GAL4*) driver to drive strong, continuous Hb expression in NB7-1 throughout its entire lineage (*en* > *hb*) and determined the number of early-born neurons generated, a quantitative measure of the length of the early competence window. To distinguish between the endogenously activated Hb protein in response to early-born identity specification and the Hb protein misexpressed from the *UAS-hb* construct, we implemented an endogenously encoded bacterial artificial chromosome (BAC) transgene that includes the *hb* genomic locus and all of its regulatory enhancers. This BAC has been modified to include a hemagglutinin epitope tag (HA) fused to Hb, and our previous work has shown that this BAC insertion is sufficient to rescue a *hb* null animal and shows the same expression pattern as the native *hb* gene [11]. Thus, we are able to use HA as a proxy for endogenous Hb expression. We co-stained Hb and Even skipped (Eve), a marker for the U motoneuron progeny of the NB7-1 lineage, and quantified the number of early-born neurons (Eve^+^HA^+^) produced in response to continuous Hb misexpression. We note that in the competence assay, the degree of neuroblast response to Hb misexpression is variable even among homologous neuroblast lineages within an individual embryo. Thus, the length of the early competence window associated with a particular genotype is represented as an average of all lineages quantified. Compared to 5.1 ± 1.0 Eve^+^HA^+^ neurons in wild type NB7-1, we found 8.0 ± 2.3 early-born neurons in *corto*^*L1*^ mutants (*p* < 0.0001). To control for any possible background mutations, we also examined transheterozygous animals of two independent loss of function alleles, *corto *^*L1*^ and *corto*^*420*^ [44] and observed consistent results, with 7.6 ± 2.7 early-born neurons generated (*p* < 0.0001) (Fig. 1B-D), indicating a prolonged early competence window. Thus, Corto plays an essential role in terminating early competence at mid-embryogenesis.

### *hb* relocation to neuroblast nuclear lamina is delayed in *corto* mutant neuroblasts

**Fig. 2:**
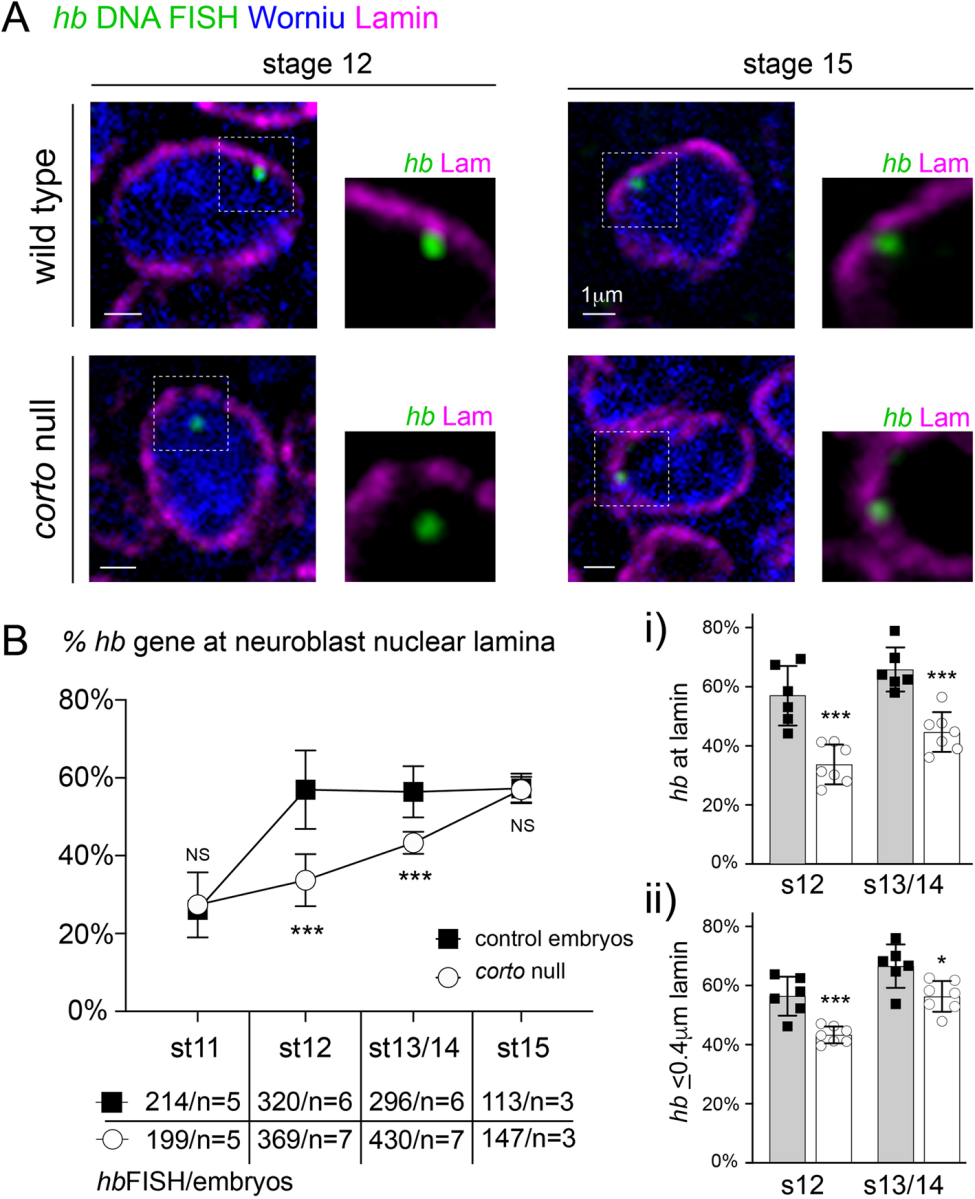
*hb* gene relocation to neuroblast nuclear lamina is delayed in *corto* mutants. **A** Single z-plane through nucleus of representative neuroblast from intact stage 12 and 15, wild type or *corto* mutant (*corto*^*L1*^) embryos, selected at the location of the brightest part of a *hb* DNA FISH signal (green spot). Immunostaining of lamin (magenta), and Worniu (pan-neuroblast, blue) shows position of *hb* loci within the three-dimensional space of neuroblast nuclei. Higher magnification of *hb* loci within neuroblast nucleus shown to the right of each FISH image; worniu is not shown for clarity. **B** Fraction of *hb* loci at the nuclear lamina (pixel-overlapping with lamin signal) quantified from wild type and *corto* mutant embryo neuroblasts across development. Each data point in line graph is the average fraction of *hb* loci at the lamina among multiple embryos (number of *hb* loci and embryos quantified shown below each stage). NS, not significant. Bar graphs to the right show mean fraction of *hb* loci either **i)** at lamin or **ii)** within 0.4µm from lamin. Each data point in bar graphs represents a single embryo. All data are represented as mean ± SD

We previously showed that the early competence window closes when the *hb* gene relocates to the neuroblast nuclear lamina at mid-embryogenesis [11, 23]. Thus, given the extension in early competence in *corto* mutants, we next examined *hb* gene positioning in *corto* mutant neuroblasts using *in vivo* immuno-DNA Fluorescence in situ Hybridization (FISH). Embryos were hybridized with a fluorescent DNA probe generated against approximately 10 kb at the *hb* gene locus and subsequently immunostained with Lamin Dm0, a B-type lamin intermediary filament that labels the nuclear envelope, and Worniu, a neuroblast-specific transcription factor. In *corto* mutant neuroblasts, we found that the proportion of *hb* gene loci at the nuclear lamina, which we defined as FISH signals that pixel-overlapped with lamin signals, was significantly reduced at mid-embryogenesis (stage 12: 56.9 ± 10.1% at lamina in wild type versus 33.7 ± 6.7% in *corto*^*−/−*^, *P* = 0.0004; stage 13/14: 56.4 + 6.6% at lamina in wild type versus 43.3 ± 2.8% in *corto*^*−/−*^, *P* = 0.0006) (Fig. 2A,B). We observed a reduction in *hb* localization to the nuclear lamina in *corto* mutant neuroblasts even when we included FISH signals that were near, but not touching the lamina (≤ 0.4µm from lamin) (Fig. 2B-ii). Interestingly, the difference between wild type and *corto* mutants diminished over time, and by late stage 15, there was no discernible difference in *hb* gene-lamina association between the genotypes (stage 15: 57.3 ± 3.8% at lamin in wild type versus 57.0 ± 3.3% in *corto *^*−/−*^ mutants, *P* = not significant) (Fig. 2B). Thus, the data show that in *corto* mutants *hb* gene relocation to the neuroblast nuclear lamina is delayed. It is worth noting that we do not observe an all-or-nothing association of the *hb* gene with the nuclear lamina, and even at stage 11, during the early competence window, ~ 25% of the *hb* gene loci are localized at the lamina. In fact, reports from others show that even genomic regions outside of lamina-associated domains are located at the periphery ~ 30% of the time [45–47], underscoring the highly dynamic and non-static nature of the genome [48]. Importantly, we observe a two-fold increase in *hb* gene association with the neuroblast nuclear lamina at mid-embryogenesis in wild type embryos that has functional consequences on neuroblast competence, and this relocation is delayed in *corto* mutants.

To confirm that the delay in *hb* gene relocation in *corto* mutants is not due to a more general delay in neuroblast divisions, we co-stained embryos with Deadpan (Dpn), a pan-neuroblast marker, and phospho-histone 3 (PH3), a marker for dividing cells. There was no difference in numbers of dividing neuroblasts at either stage 12 or 14. Embryonic neuroblasts gradually divide more slowly as neurogenesis progresses, and this dynamic was unchanged in *corto* mutants, suggesting that neuroblast proliferation is progressing normally (Figure S1).

### Normal Hb temporal dynamics in *corto* mutant neuroblasts

**Fig. 3:**
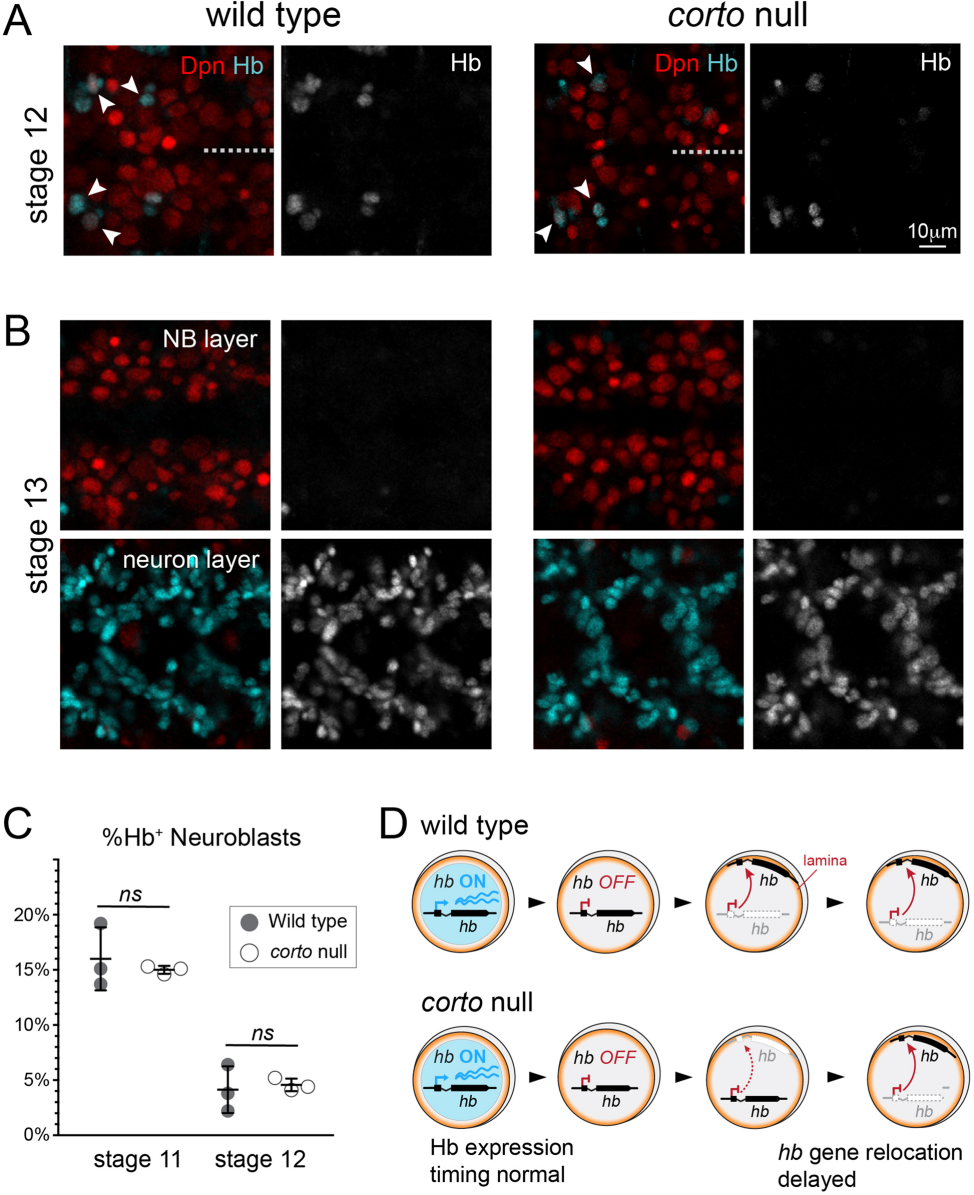
Temporal dynamics of Hb repression in neuroblasts is not affected in *corto* mutants. **A** Ventral view of VNC wild type or *corto* mutant (*corto*^*L1*^) embryos at late stage 12, when the last few neuroblasts in the thoracic segments still express Hb (cyan), but the rest, including all abdominal neuroblasts, are no longer Hb^+^. Neuroblasts are marked by pan-neuroblast marker, Dpn (red). **B** At stage 13, Hb is no longer detectable in any neuroblasts (top panels) but can be detected in neurons, deeper in the VNC (bottom panels). Hb shown alone in grayscale in A and B. **C** Quantification of fraction of neuroblasts expressing Hb at stage 11 and stage 12 comparing wild type and *corto* mutant embryos, showing similar decrease in Hb neuroblast expression over time. **D** Schematic diagram summarizing results

While genes localized at the nuclear lamina are typically in a repressed or silenced state, actively transcribed genes are often localized interiorly [49]. If the delay in *hb* gene relocation to the neuroblast nuclear lamina in Corto mutant embryos is due to prolonged *hb* transcription, we would expect a concomitant increase in the number of early-born neurons produced. By immunostaining for Eve, we did observe on occasion ectopic early-born neurons, but the occurrence was rare (2 Eve^+^Hb^+^ neurons in wild type compared to an average 2.1 in *corto*^*L1/420*^ mutants) (Figure S2). *Drosophila* embryo development is highly stereotyped, allowing us to use morphological characteristics to compare stage-matched animals of different genotypes, and we found no qualitative differences in the timing of Hb repression in neuroblasts between wild type and *corto* mutants (Fig. 3A). By late stage 12, Hb is expressed by only a few neuroblasts in the thoracic segments (Fig. 3A, arrowheads) and is not detectable in any neuroblasts at stage 13, but rather is expressed in the neuronal layer, deeper in the embryo (Fig. 3B). Upon quantifying the proportion of Hb^+^ neuroblasts between stage 11 and 12, when Hb is rapidly being repressed across the neuroblast population, we measured no difference between wild type and *corto* mutants, indicating Hb was downregulated in a timely manner (Fig. 3C). Further arguing against a strong role for Corto as a *hb* transcriptional repressor, we found that Corto overexpression in neuroblasts had no effect on the production of early-born neurons (Figure S3), in contrast to *hb’s* known transcriptional repressor, Svp, which reduces the number of early-born neurons upon overexpression [21]. Thus, the impaired *hb* gene-lamina relocation phenotype in *corto* mutant neuroblasts we observed at stages 12–14 (7-11 h after egg lay, AEL), is well *after hb* is already transcriptionally downregulated, and the relocation phenotype is not due to prolonged *hb* transcription (prior to 7 h AEL) that keeps it in the nuclear interior.

### Psc and Corto genetically interact to restrict the early competence window

**Fig. 4:**
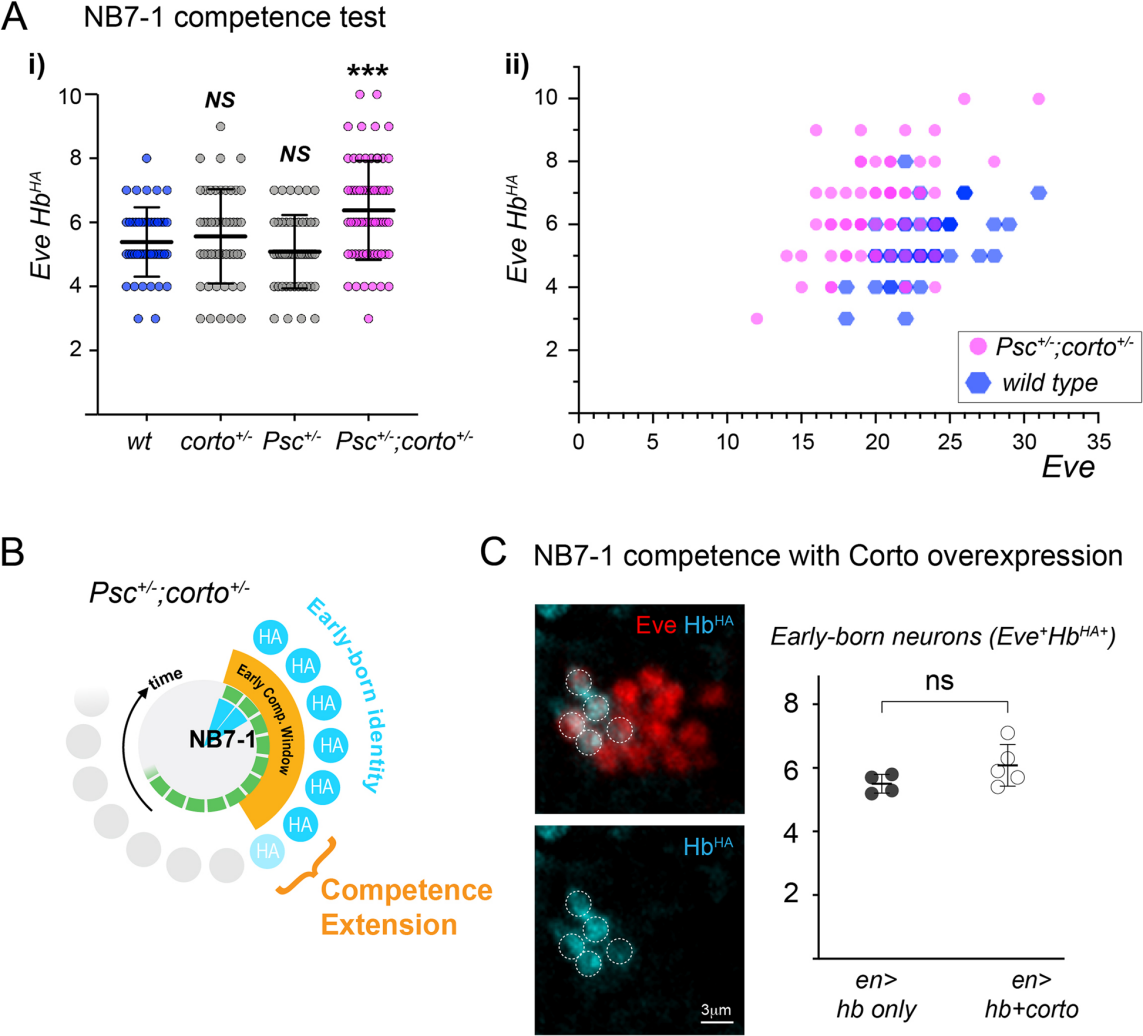
Corto and Psc genetically interact to close the early competence window. **A** i), NB7-1 lineage early competence assay comparing *wild type*, *corto*^*L1/*+^, *Psc*^*h27/*+^, or *corto *^*L1/*+^*;Psc*^*27/*+^ transheterozygous embryos. Bars represent mean ± SD. ii), Scatterplot of *wild type* and *corto *^*L1/*+^*;Psc*^*27/*+^ data, showing the number of Eve^+^HA^+^ (early-born) neurons relative to the total number of Eve neurons within an individual NB7-1 lineage. Each data point represents competence data of a single NB7-1 lineage quantified from three to four embryos per genotype. **B** Schematic circular diagram depicting results. **C** Early competence window test with and without co-overexpression of Corto. Example NB7-1 lineage shown for Hb and Corto co-misexpressed by *en-Gal4* driver, stained for Hb^HA^ (cyan) and Eve (red). Quantification shown to the right. Each data point represents the average of at least twelve neuroblast lineages from a single embryo. Bars represent mean ± SD

Corto has been reported to interact with PcG chromatin factors to maintain gene repression [29, 40]. In particular, Corto shares many binding sites on polytene chromosomes with Psc [40, 42], which plays a central role in PRC1 silencing activity [27]. We asked whether Corto and Psc genetically interact to terminate competence by testing the early competence window in transheterozygous mutant embryos. We examined embryos stages 14–16, the tail end of the NB7-1 lineage, and found that while neither *Psc* heterozygotes or *corto* heterozygotes alone have a competence phenotype (P value not significant), *Psc*^*h27/*+^;*corto*^*L1/*+^ transheterozygous animals have prolonged early competence (Fig. 4A) (*Psc*^*h27/*+^;*corto*^*L1/*+^ 6.4 ± 1.5 vs. wild type 5.4 + 1.1, *P* = 0.0004). Given the variability in response to Hb misexpression even among homologous neuroblasts within the same embryo, we additionally displayed the data as a scatterplot, showing the number of early-born neurons relative to Eve^+^ neurons, representing the total cells in the lineage upon Hb misexpression (Fig. 4A-ii). *Psc*^*h27/*+^;*corto*^*L1/*+^ transheterozygous neuroblasts generated, overall, a higher fraction of early-born neurons than wild type neuroblasts with similar numbers of Eve^+^ cells, indicating increased competence among neuroblasts with comparable levels of Hb misexpression. We note that for the stages analyzed, the number of Eve^+^ cells is higher in older embryos, as expected (stage 14: 18.7 ± 2.9 Eve^+^ cells at stages 14, *n* = 68 lineages from 5 embryos, vs stage15/16: 23.0 ± 3.6, *n* = 136 lineages from 8 embryos, *p* < 0.0001), whereas the number of Eve^+^HA^+^ (early-born) neurons did not correlate with stage (stage 14: 5.9 ± 1.5 Eve^+^HA^+^ neurons vs stage 15/16: 5.6 ± 1.4, not significant). This confirms that all embryos sampled had completed production of early-born neurons. Together, we conclude that Psc and Corto genetically interact to close the competence window to specify early-born neurons.

In contrast, overexpression of Corto did not have any effect on competence. While wild type animals had an average of 5.5 ± 0.3 Eve^+^HA^+^ neurons upon Hb misexpression (68 hemisegments quantified from *n* = 4 embryos), co-overexpression of Corto did not yield a statistically different result, 6.1 ± 0.3 Eve^+^HA^+^ neurons (86 hemisegments quantified from *n* = 5 embryos) (Fig. 4C). If Corto acts in neuroblasts primarily through its interactions with the PcG complex, perhaps the stoichiometry or activity level of one or more PcG complex subunits is rate-limiting.

### Corto does not act to maintain repression of Hb or the Hox gene, Abdominal B, in neuroblasts

**Fig. 5:**
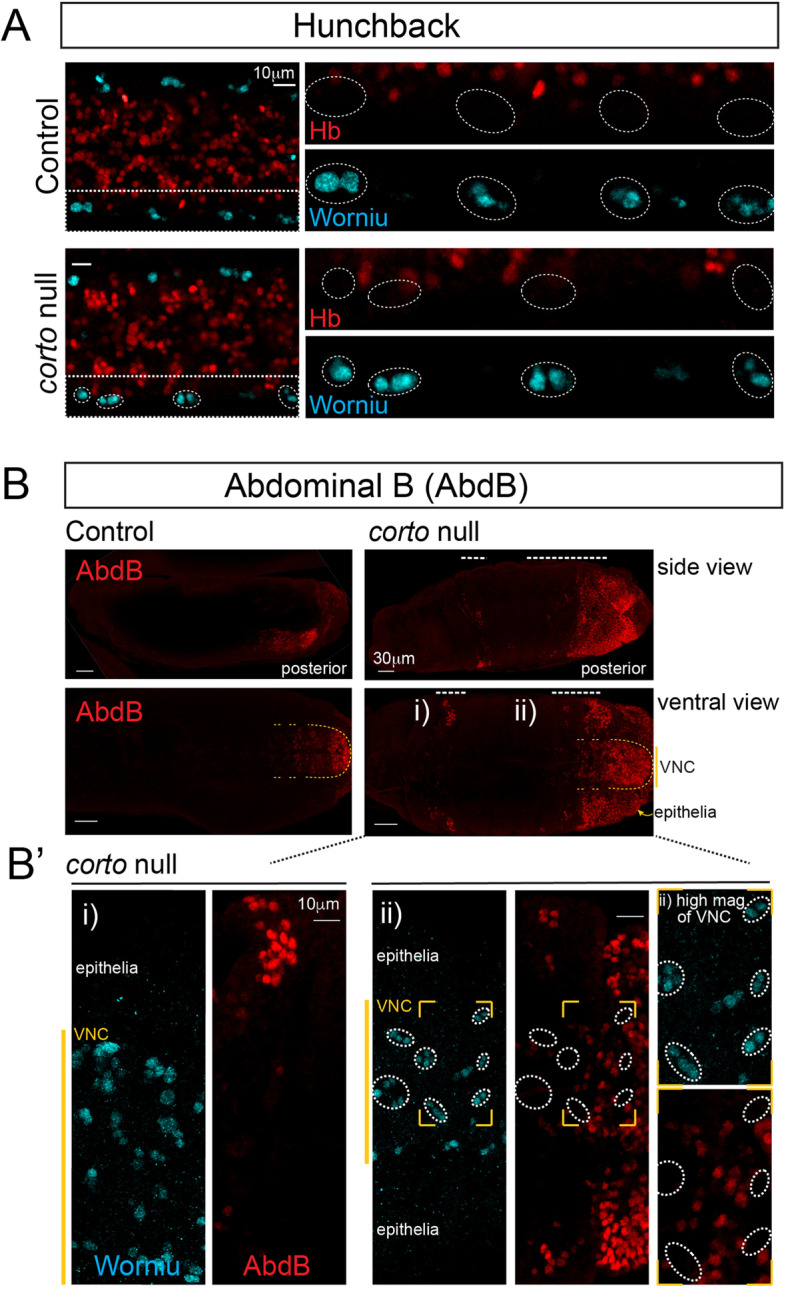
Hb and Abd-B are not derepressed in neuroblasts in late stage *corto* mutant (*corto*^*420*^) embryos. **A** VNC of control (*corto*^+/-^) or *corto* mutants at stage 15, stained with Worniu (pan-neuroblast marker, cyan) and Hb (red). Dashed box region shown as separate Worniu and Hb panels to the right. **B** Control and *corto* mutant whole embryos at stage 15 are shown as side and ventral views, stained with Abd-B. Dashed white lines at the top show areas of derepressed Abd-B. Yellow dashed line demarcates VNC region. **B’** Higher magnification of regions labeled i) and ii) in ventral view of *corto* mutant in B. Yellow vertical line show VNC region. Area within yellow box in ii) are shown in higher magnification to the right

**Fig. 6:**
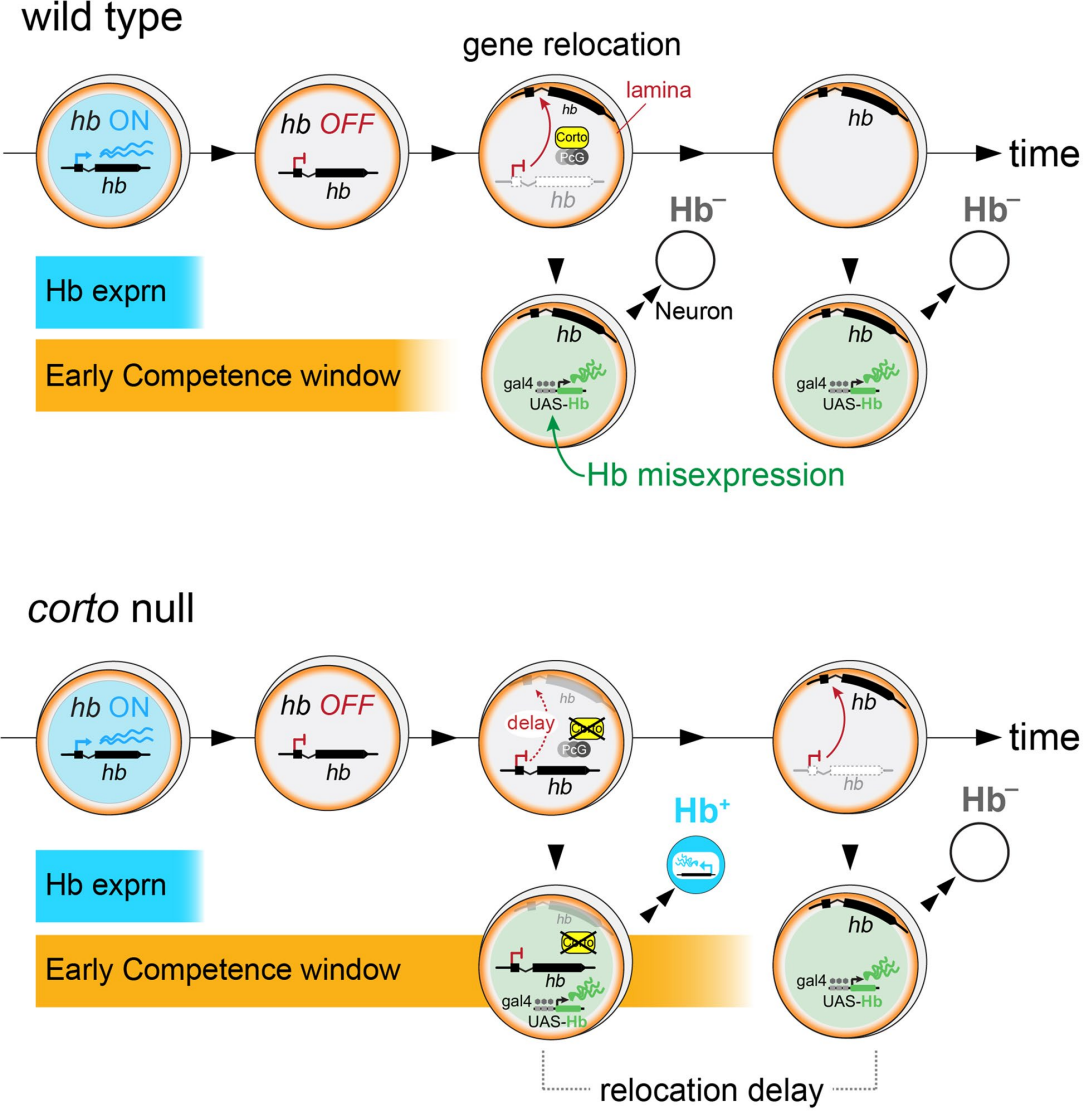
Summary model. In both wild type and *corto* mutant neuroblasts, *hb* transcriptional dynamics are largely normal (endogenous *hb* depicted in blue). Much after *hb* repression, *hb* gene relocation to the neuroblast nuclear lamina terminates the early competence window. Loss of Corto, which genetically interacts with PcG, delays this relocation and extends early competence (misexpressed Hb depicted in green)

PcG factors have been well studied for their roles in maintenance of target gene repression. As an enhancer of PcG, Corto has been reported to play a similar role in the repression of Abd-B, a known PcG Hox target gene, and loss of Corto results in its derepression [41]. Our recent study [23] showed that the *hb* intron region is a strong PcG target site, and loss of PRC1 impairs *hb* gene-lamina relocation. Interestingly, however, loss of PcG does not result in Hb derepression in neuroblasts even in late stage embryos. Moreover, even the most severe loss of function mutants for PRC1 (*Psc-Su(z)2*^*P3C*^) and PRC2 (maternal and zygotic mutants of *extra sex combs*, *esc*^*mat/zyg*^), did not show any neuroblast derepression of Hb in late-stage embryos [23], raising the possibility that PcG factors may not function in transcriptional repression in this cell type. We thus examined Hb expression in late-stage *corto* mutant embryos to determine whether Hb becomes derepressed. Similar to the PRC1 mutants, we also did not find Hb derepressed in neuroblasts of late stage *corto* mutant embryos (Fig. 5A). While we did observe Abd-B derepression in *corto* mutants, consistent with previous observations [41], surprisingly, the derepression was limited to the epithelia, and we did not find Abd-B derepressed in neuroblasts (Fig. 5B-C). In summary (Fig. 6), our results show that Corto is required for the timely relocation of the *hb* gene to the neuroblast nuclear lamina and genetically interacts with PRC1 to terminate early competence. However, it does not play a role in maintaining *hb* gene repression, and in a departure from its known function, it does not play a role in repression of its known target *Abd-B*, within neuroblasts.

## Discussion

Here we identify Corto as a regulator of neuroblast early competence in the *Drosophila* embryo. Loss of Corto results in a delay in *hb* gene relocation to the nuclear lamina in neuroblasts and an extension in the early competence window. While the low numbers of extra early-born neurons found in a subset of neuroblast lineages in *corto* mutant embryos could indicate an occasional extension of neuroblast Hb expression, we did not observe any measurable changes to Hb transcriptional dynamics in *corto* mutants across the neuroblast population. In contrast, the delay in *hb* gene relocation was robust across neuroblasts and occurred substantially after the *hb* gene was already repressed and thus not a result of prolonged transcriptional activity.

In the fly embryo, neuroblasts transiently express a series of temporal identity factors which confer specific traits to the neurons descended from each division. Hb, the first of the temporal identity factors, specifies early-born identity, which includes endogenous activation of the *hb* gene within the postmitotic neuron. Misexpression of Hb directly in the postmitotic neuron cannot induce *hb* transcription [11, 12]. The molecular identity of the neuron, including its ability to activate and sustain *hb* transcription endogenously, must be established within the neuroblast progenitor and subsequently inherited. Thus, the neuroblast “primes” the future transcriptional program that is activated by the neural progeny. Critically, the neuroblast’s potential to generate an *hb*-transcribing early-born neuron ends not when *hb* is transcriptionally repressed within the neuroblast, but rather several divisions later when the *hb* gene relocates to the nuclear lamina. Upon relocation, *hb* becomes refractory to activation within the descendent neuron, thus closing the early competence window. Corto is required for this heritable silencing through *hb* gene relocation to the neuroblast nuclear lamina. However, relocation in *corto* mutants is only delayed, not completely blocked, as the *hb* gene gradually increases association with the lamina, perhaps due to compensation by PcG factors with which it interacts. Consistent with this notion, overexpression of Corto alone did not shorten the early competence window.

We have focused on *hb* as a model gene to study mechanism of neuroblast competence regulation, as it is the only universally expressed gene among an otherwise diverse population of early-born neurons, but it is not clear what role, if any, Hb itself plays in the postmitotic, early-born neurons of the VNC. RNAi-mediated knockdown of Hb in NB7-1 neural progeny does not alter neuronal morphology or affect larval locomotor velocity [50]. We speculate that *hb* is only one component of a broad transcriptional program in postmitotic neurons established by the nuclear architecture of the neuroblast upon specification of early-born identity, and *hb* transcription may reflect its epigenetic state, rather than a requirement for function in these neurons. In the postembryonic brain, however, Hb in postmitotic neurons has been shown to play an important role in sexually dimorphic interneuron neurite morphology [51], and a recent study established a functional role for Hb in larval Pair1 neuron in synapse number, connectivity, and behavior [52]. It would be important for future studies to determine gene-lamina interactions in neuroblasts on a genome-wide scale and relate these lamina-associated genes to neuroblast competence, gene expression and neuronal function. In this context, identifying trans-acting factors of nuclear architecture and understanding the mechanisms of their function is a critical area requiring further study. Here we have found Corto, which impacts *hb* gene relocation without affecting *hb* transcriptional dynamics.

Though Corto has been shown to participate in PcG-mediated silencing [40, 41], we did not observe any Hb derepression in neuroblasts in later embryonic stages, consistent with our recent observations for PcG [23]. While PcG factors bind the *hb* gene locus and are required for *hb* gene relocation in neuroblasts, they do not function in regulation of *hb* transcriptional repression, a departure from their well-known roles in maintenance of target gene repression. Further supporting the lack of a repressive role in neuroblasts, overexpression of Corto did not reduce the number of early-born neurons, which is in contrast to what has been reported for Svp, *hb’s* established transcriptional repressor [21, 22]. Additionally, PcG and Corto have been shown to be required for repression of Abd-B, the PcG Hox target gene, and similar to observation by others, we found Abd-B to be derepressed in *corto* mutants. Interestingly, however, while we found Abd-B derepression in the epithelia, we did not observe this in neuroblasts. Perhaps this suggests that PcG/Corto have a unique role in neuroblasts by facilitating changes to their nuclear architecture, a function independent of their better known roles in transcriptional repression. Further studies are necessary to investigate a more general role of PcG/Corto in neuroblasts comparing genome architecture and transcriptional regulation, and understand how such activity underlie neuroblast competence to determine neuronal identity and function.

## Conclusions

Increasing evidence points to an important role for three dimensional organization of genome architecture in regulating transcriptional and competence states of progenitors during animal development [53, 54]. We have identified Corto, an ETP class chromatin factor, as a new regulator of neuroblast competence. Corto is required for the timely relocation of the *hb* gene to the neuroblast nuclear lamina and genetically interacts with Psc to close the early competence window. In neuroblasts, loss of Corto does not impact *hb* transcriptional dynamics nor does it cause derepression of Abd-B, a departure from its known role in Abd-B repression. By identifying new chromatin regulators of competence, our results provide further mechanistic insights into how progenitor competence is regulated *in vivo*. Together, the results show that multiple, distinct repressive mechanisms sequentially operate in a step-wise fashion as neuroblasts age over time to terminate neuroblast competence to specify early-born neural identity.

## Supplementary Information

**Additional file 1.**

## Data Availability

No datasets or new fly stocks were generated. Fly stocks used in this manuscript are available from public stock centers or will be provided upon request to corresponding author.
