# Supplementary material for: Enhancer of trithorax/polycomb, Corto, regulates timing of hunchback gene relocation and competence in Drosophila neuroblasts
Source: Neural Dev. 2022 Feb 17;17:3. doi: 10.1186/s13064-022-00159-3 (PMC8855600; doi:10.1186/s13064-022-00159-3)
Supplement: Supplementary file 1 — Additional file 1. [file 13064_2022_159_MOESM1_ESM.docx]

**Supplemental Information**

Manuscript: Enhancer of trithorax/polycomb, Corto, regulates timing of *hunchback* gene relocation and competence in *Drosophila* neuroblasts.

Hafer T.L., Patra S, Tagami D, and Kohwi M.


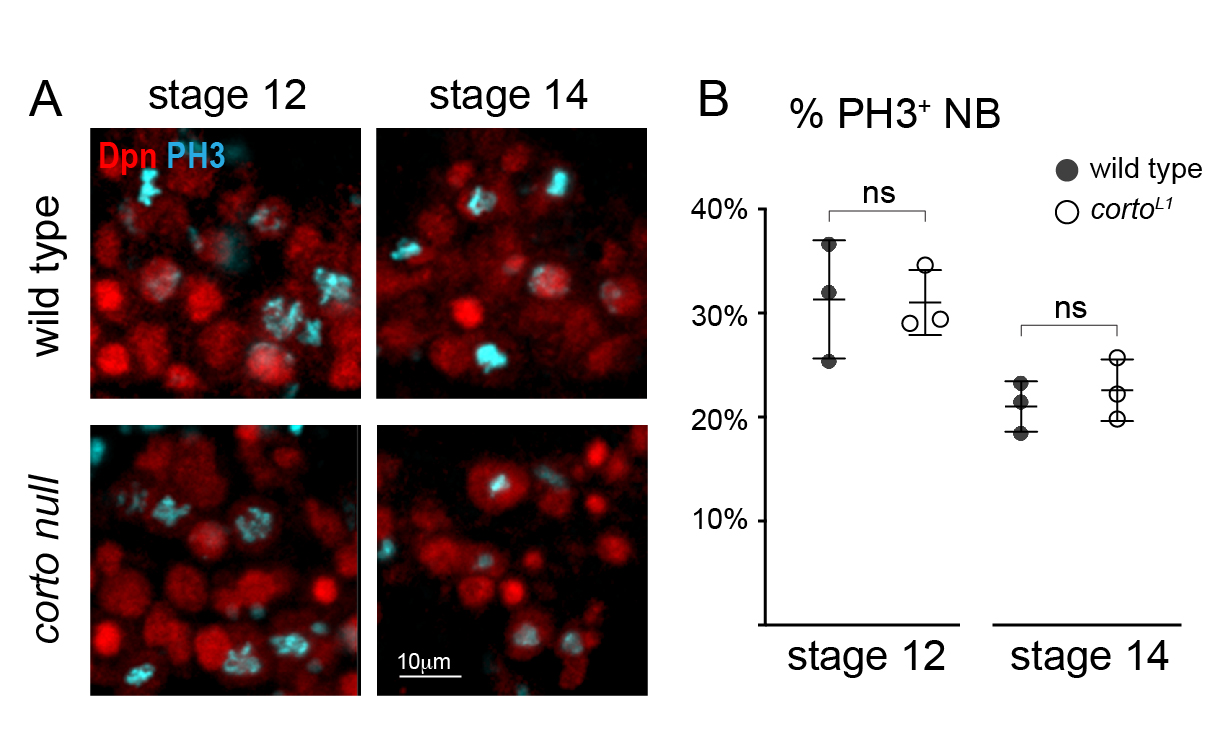


**Supplemental Figure 1 (Supplement to Figure 2)**

**A.** Wild type and *corto* null embryos (*corto^L1^*) were stained with Dpn (pan-neuroblast marker, red) and phosphohistone 3 (PH3, cyan). Representative hemisegments shown at stage 12 and stage 14.

**B.** Quantification of PH3^+^ neuroblasts in wild type and *corto* mutant embryos. Each data point represents a single embryo. Errors bars show mean ­+ SD.


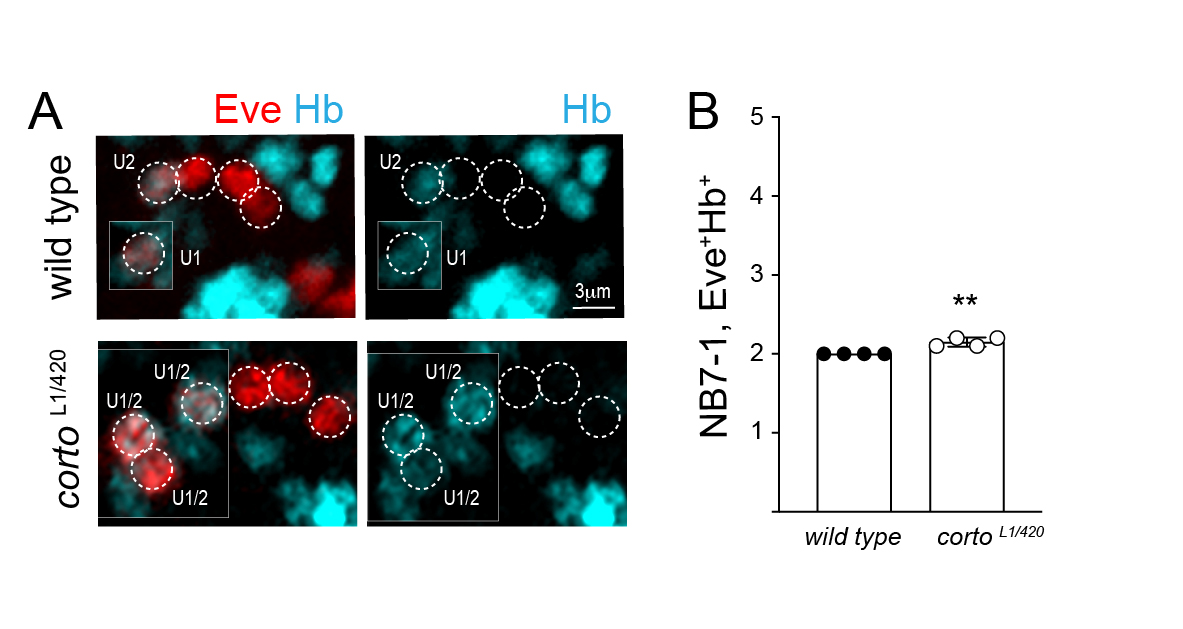


**Supplemental Figure 2 (Supplement to Figure 3)**

**A.** NB7-1 lineage U motoneuron progeny (Eve^+^) co-stained with Hb (early-born neurons). Example of extra early-born neuron (U1/2) shown in *corto* null (*corto^L1/420^*) embryo.

**B.** Quantification of early-born neurons from NB7-1 in wild type and *corto* mutant embryos.


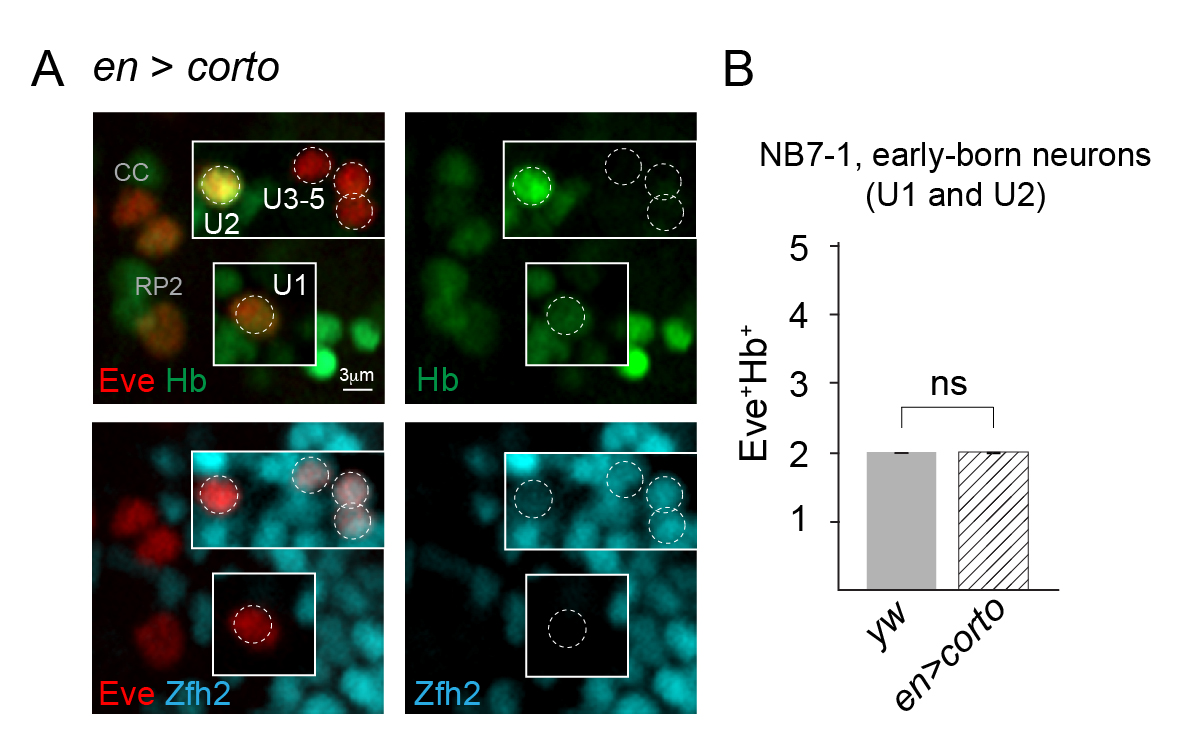


**Supplemental Figure 3 (Supplement to Figure 3)**

**A.** The *engrailed*-gal4 driver was used to overexpress corto (*en>corto*) in row 6,7 neuroblasts, which include NB7-1. Eve stain (red) shows no difference in NB7-1 progeny (U motoneurons); U1-2 are Hb^+^ (green) and U2-5 are Zfh2^+^ (cyan).

**B.** Quantification of U1/2, early-born neurons.
